# *Natrarchaeobiusversutus* sp. nov. and *Natrarchaeobius oligotrophus* sp. nov., chitinotrophic natronoarchaea from hypersaline soda lakes, and functional genome analysis of the *Natrarchaeobius* species

**DOI:** 10.3389/fmicb.2025.1640521

**Published:** 2025-07-30

**Authors:** Adolf S. Tulenkov, Alexander G. Elcheninov, Dimitry Y. Sorokin

**Affiliations:** ^1^Winogradsky Institute of Microbiology, Research Centre of Biotechnology RAS, Moscow, Russia; ^2^Moscow Center for Advanced Studies, Moscow, Russia; ^3^Department of Biotechnology, Delft University of Technology, Delft, Netherlands

**Keywords:** natronoarchaea, soda lakes, *Natrarchaeobius*, chitin, chitinase, glycoside hydrolases

## Abstract

Polysaccharide-degrading natronoarchaea have been poorly studied to date. However, over the past decade, significant progress has been made in understanding their diversity and metabolic potential. In this study, two natronoarchaeal strains, enriched from oxic sediment samples of the soda lakes of Wadi an Natrun in Egypt (AArcel7) and Kulunda steppe in Russia (A-rgal3), were characterized. Strain AArcel7 was enriched with amorphous cellulose, while strain A-rgal3 dominated an enrichment culture using rhamnogalacturonan. Cells of both strains are polymorphic, from motile flat rods to nonmotile cocci. They are aerobic heterotrophs that are able to grow on chitin and several other carbohydrates. Both strains thrive within a salinity range of 2.5 to 4.5 M total Na^+^, with optimal growth at 3.5–4 M, and are moderately alkaliphilic with an optimum pH at 8.5–9.0 (AArcel7) and 9.2–9.5 (A-rgal3). Genome-based phylogenetic analysis demonstrated that these isolates form a new species lineage in the chitin-specialized genus *Natrarchaeobius*. An in-depth study of *Natrarchaeobius* genomes allowed us to identify several genes that potentially enable them to hydrolyze chitin and to metabolize N-acetylglucosamine (GlcNAc), which has not been investigated previously in the chitin-utilizing natronoarchaea. Based on physiological, phylogenetic, and genomic analyses, strains AArcel7 and A-rgal3 are suggested to form a novel species, *Natrarchaeobius versutus* sp. nov., with AArcel7^T^ (DSM 119357 = UNIQEM U973) as the type strain. Furthermore, strain AArcht7^T^, formerly classified as the type species of the genus *Natrarchaeobius*, is proposed to be reclassified as *Natrarchaeobius oligotrophus* (DSM 119677 = UNIQEM U967).

## Introduction

Hypersaline environments such as inland salt lakes and marine solar salterns, where the salt concentrations can reach the saturation point, host diverse communities of halophilic microorganisms, often dominated by extremely halophilic archaea of the class *Halobacteria* (Oren, 2013). Some hypersaline ecosystems, including soda lakes or solonchaks, are further distinguished not only by elevated concentrations of NaCl but also by high concentrations of sodium carbonate and diminished concentrations of divalent cations. These conditions result in the formation of haloalkaline environments (pH range of 9–11), which are inhabited by haloalkaliphiles (or natronophiles). Until recently, knowledge on the metabolic versatility of halo(natrono)archaea was mostly limited to aerobic or facultatively anaerobic organoheterotrophs, utilizing simple compounds such as sugars, amino acids, and organic acids (Andrei et al., 2012). However, hypersaline lakes are rich in organic polymers produced by algae, arthropods, and crustaceans (Dana et al., 1990; Harper et al., 2003; Dadheech et al., 2013; Mengistou, 2016). Furthermore, such open systems can also trap complex organic matter of a terrigenic origin. Recent decades have revealed that extremely (halo)alkaliphilic archaea are capable of utilizing complex polymeric substrates, including chitin (Sorokin et al., 2015), cellulose (Sorokin et al., 2015; Elcheninov et al., 2023), and other polysaccharides (Sorokin et al., 2022).

Chitin is one of the most abundant types of polymer not only in freshwater and marine environments, but also in hypersaline lakes and salterns (produced *en masse* there by the brine shrimp *Artemia*). Nevertheless, despite the fact that endo-chitinase and β-N-acetyl-hexosaminidase genes have already been identified in haloarchaeal metagenomes from Siberian soda lakes (Vavourakis et al., 2016) and growth on chitin has been demonstrated in the chitin-specialized haloarchaeal genera *Natrarchaeobius*, *Salinarchaeum,* and *Halomicrobium* (Sorokin et al., 2015; Sorokin et al., 2019; Minegishi et al., 2017; La Cono et al., 2020), there is still a lack of emphasis on the precise mechanisms of chitin utilization in extremely halophilic archaea. Another polymers occurring in soda lakes are uronic acid-containing polysaccharides (e.g., pectin, rhamnagalactouronan, and some others). Despite the presence of acidic polysaccharide-hydrolyzing lyases of the PL superfamily in the genomes of some haloarchaea (Sorokin et al., 2022), there is still no evidence that these microorganisms can hydrolyze and further utilize such polysaccharides as growth substrates.

In this study, we report on the isolation and phenotypic characterization of two closely related natronoarchaeal strains, AArcel7^T^ and A-rgal3, isolated from hypersaline alkaline lakes, and propose to classify them as a novel species within the genus *Natrarchaeobius*. In addition, strain AArcht7^T^, described previously as a member of the type species of *Natrarchaeobius*, is proposed to be reclassified into a separate species within this genus. The taxonomy data are also supplemented with the functional genome analysis focused on encoded chitin-degrading enzymes and proteins probably involved in its monomer catabolism.

## Materials and methods

### Culture isolation and cultivation

Basic mineral medium used for enrichment and routine cultivation included 3 M NaCl + sodium carbonate/bicarbonate buffer (1 M on the basis of total Na^+^, pH 10), 5 g L^−1^ KCl, and 1 g L^−1^ K_2_HPO_4_. After autoclaving at 120°C for 20 min, it was supplemented with 4 mM NH_4_Cl, 1 mM MgSO_4_, and 1 mL each of vitamin mix and acidic trace metal solution (Pfennig and Lippert, 1966; Pfennig and Trüper, 1992). The final pH was 9.5. Solid medium was prepared by mixing 3 parts of liquid medium and 2 parts of 4.5% sterilized, washed agar at 55°C. To compensate for salinity dilution by agar, solid NaCl was added to the liquid medium before mixing it with agar. The pH range for growth was assayed with cellobiose at 4 M total Na^+^. For the pH range from 6 to 8, a combination of HEPES and K-P buffers (50 mM each) + 4 M NaCl was used with an overlap with 3.9 M NaCl/0.1 M NaHCO_3_ for pH 8. For experiments at higher pH values (up to 10.5), the medium contained 3 M NaCl and 1 M Na^+^ provided as carbonate/bicarbonate in varying proportions. Actual pH values were monitored throughout the experiment. Total Na^+^ range for growth was investigated at pH 9.5 with cellobiose as substrate, retaining the NaCl: Na (as carbonates) ratio 3:1. Growth substrate utilization tests were performed in liquid medium at pH 9.5, 4 M total Na^+^ and 35°C with 1 g L^−1^ (for sugars and polysaccharides) or 10 mM for other compounds. Cultures were incubated on a rotary shaker at 150 rpm, and growth was monitored by measuring OD_600_ in comparison to a control without added substrate. In case of insoluble polysaccharides, 1 mL samples were centrifuged for 10 s at 2,000 rpm in 2 mL Eppendorf tubes, and the top 0.5 mL layer was used for measurements. Additionally, chitin degradation was assessed visually, based on the formation of a uniform opaque halo around colonies during cultivation on solid medium with amorphous chitin and disappearance of insoluble chitin forms (both amorphous and native from shrimps) in liquid cultures. Potential for anaerobic growth was tested with cellobiose, either alone for fermentative growth, or in the presence of nitrate, sulfur, thiosulfate, DMSO, and fumarate (5 mM each). The tests were performed in 12 mL serum bottles closed with butyl rubber stoppers, with 10 mL medium made anoxic by 3 cycles of sterile argon flushing-evacuation. In the case of sulfur and thiosulfate, 0.5 mM sulfide was added as a reductant.

Cell morphology was examined with phase contrast and electron microscopy. For the latter, the cells were prepared as described previously (Sorokin et al., 2019). Briefly, the cells were fixed with paraformaldehyde (3% v/v final) for 2 h at 4°C, resuspended in fresh buffer containing 4 M NaCl at pH 7 and either positively contrasted with 1% uranyl acetate for the direct transmission electron microscopy or postfixed with OsO_4_ (2% w/v in 4 M NaCl), dehydrated, and epoxidated for thin section electron microscopy. Membrane polar lipids and menaquinones were extracted from the freeze-dried cells of strain AArcel7 and identified by HPLC-MS chromatography as described previously (Bale et al., 2019).

### Genome sequencing, assembly, and initial annotation

Genome of strain AArcel7^T^ was sequenced previously (Elcheninov et al., 2023) and annotated using IMG/M pipeline v.4.16.5 (Markowitz et al., 2014). Extracted genomic DNA of strain A-rgal3 was used for library preparation (150 bp paired-end), and whole genome sequencing (Illumina NovaSeq 6000) was performed by Novogene (United Kingdom). Obtained reads were filtered by quality and length using CLC Genomic Workbench v.10 (Qiagen). The genome was assembled in two stages. Initially, trusted contigs were generated with Unicycler v.0.4.9 (Wick et al., 2017). Further SPAdes v.3.15.4 (Bankevich et al., 2012) was used: “isolate” mode with “trusted contigs” option. Contigs with a length below 500 bp or with low coverage were excluded from the final assembly. Completeness and contamination levels of the final assembly were estimated using CheckM v.1.2.2 with the *Archaea* marker set (Parks et al., 2015). The genome was annotated using NCBI Prokaryotic Genome Annotation Pipeline (PGAP) v.6.7 (Tatusova et al., 2016).

### Phylogenetic and functional genome analyses

Alignment of 16S rRNA gene sequences from *Natrialbacea* type strains was done with MAFFT v.7.490 and the E-INS-i algorithm (Katoh and Standley, 2013). Phylogenetic tree reconstruction based on comparison of 16S rRNA genes was done in IQTree2 v.2.3.5 (Minh et al., 2020) with automatically determined best model (TIM + F + R4) and 1,000 ultrafast bootstrap replicates. Phylogenomic analysis was performed with a set of archaeal marker proteins of the “ar122” set (Rinke et al., 2021) for the *Natrialbacea* type strains. Amino acid sequences of marker proteins were found, aligned, and concatenated with gtdb-tk v.1.7.0 (Chaumeil et al., 2019). The resulting alignment was trimmed with trimAL v.1.4.1 (with-automated1 option) (Capella-Gutiérrez et al., 2009). To reconstruct the maximum likelihood phylogenetic tree, RAxML v.8.2.12 (Stamatakis, 2014) was used with 1,000 rapid bootstraps and PROTGAMMAILG substitution model. All phylogenetic trees were visualized in the iTOL v.6 (Letunic and Bork, 2024).

Average nucleotide identities (ANI) were calculated with the OrthoANI tool (Lee et al., 2016). Average amino acid identities (AAI) were calculated using the aai-matrix.bash and aai.rb scripts from the enveomics collection (Rodriguez-R and Konstantinidis, 2016). To estimate digital DNA–DNA hybridization (dDDH), GGDC v.3.0 was used (Meier-Kolthoff et al., 2022). ANI and AAI data were visualized in Python3 using the seaborn (Waskom, 2021) and the matplotlib packages (Hunter, 2007).

Carbohydrate-active enzymes (CAZymes) were searched using dbCAN v.4 (Zheng et al., 2023) with HMMER (Mistry et al., 2013) and Diamond (Buchfink et al., 2015) tools. Predicted enzymes were further checked by BLAST against the Swiss-Prot database. Visualization of identified CAZymes was performed using the R programming language and the ggplot2 package (Wickham, 2016). Domain structures of chitinases were detected using dbCAN_sub output and InterPro Scan (Pfam database) (Blum et al., 2025). The synteny of chitinase-encoding genes, along with domain organization of chitinases, was visualized using the gggenes package (Wilkins, 2023).

## Results and discussion

### Isolation and phenotypic properties of pure cultures

Strain AArcel7^T^ was enriched with amorphous cellulose as sole substrate from mixed surface sediments of several Wadi an Natrun hypersaline alkaline lakes in the Libyan Desert (Egypt). In contrast to the dominant cellulotrophic natronoarchae belonging to *Natronobiforma cellulositropha* (Sorokin et al., 2018), AArcel7^T^ had insignificant amorphous cellulose clearance around its colonies and did not grow back on amorphous cellulose in liquid culture, while routinely cultured on cellobiose. Strain A-rgal3 was enriched from a mixture of oxic sediments samples from 3 hypersaline soda lakes in Kulunda Steppe (Altai region, Russia) taken in 2017. The enrichment targeted aerobic pectinolytic natronoarchaea, which, so far, eluded detection in hypersaline habitats. There was only a very weak growth on apple pectin, and A-rgal3 was eventually isolated from an offshoot on rhamnogalacturonan plates, forming large pink spreading colonies on a hypersaline agar at pH 9.5 supplemented with rhamnogalacturonan (Megazyme). However, growth in liquid culture with the galacturonate-based polysaccharides (pectins, rhamnogalacturonan, and polygalacturonate) and the galacturonate monomer was either marginal or absent, and eventually, the culture was cultivated on cellobiose. The purity of the isolates was confirmed by sequencing of its 16S rRNA gene as well as whole genome sequencing. The strains have been preliminarily identified using BLAST search with 16S rRNA gene sequences of both strains as queries against the NCBI core_nt database. The two isolates were closely related to each other (>99.8% sequence identity) and potentially represented a new species lineage within the soda lake chitinotrophic genus *Natrarchaeobius* (Supplementary Figure S1).

**Figure 1:**
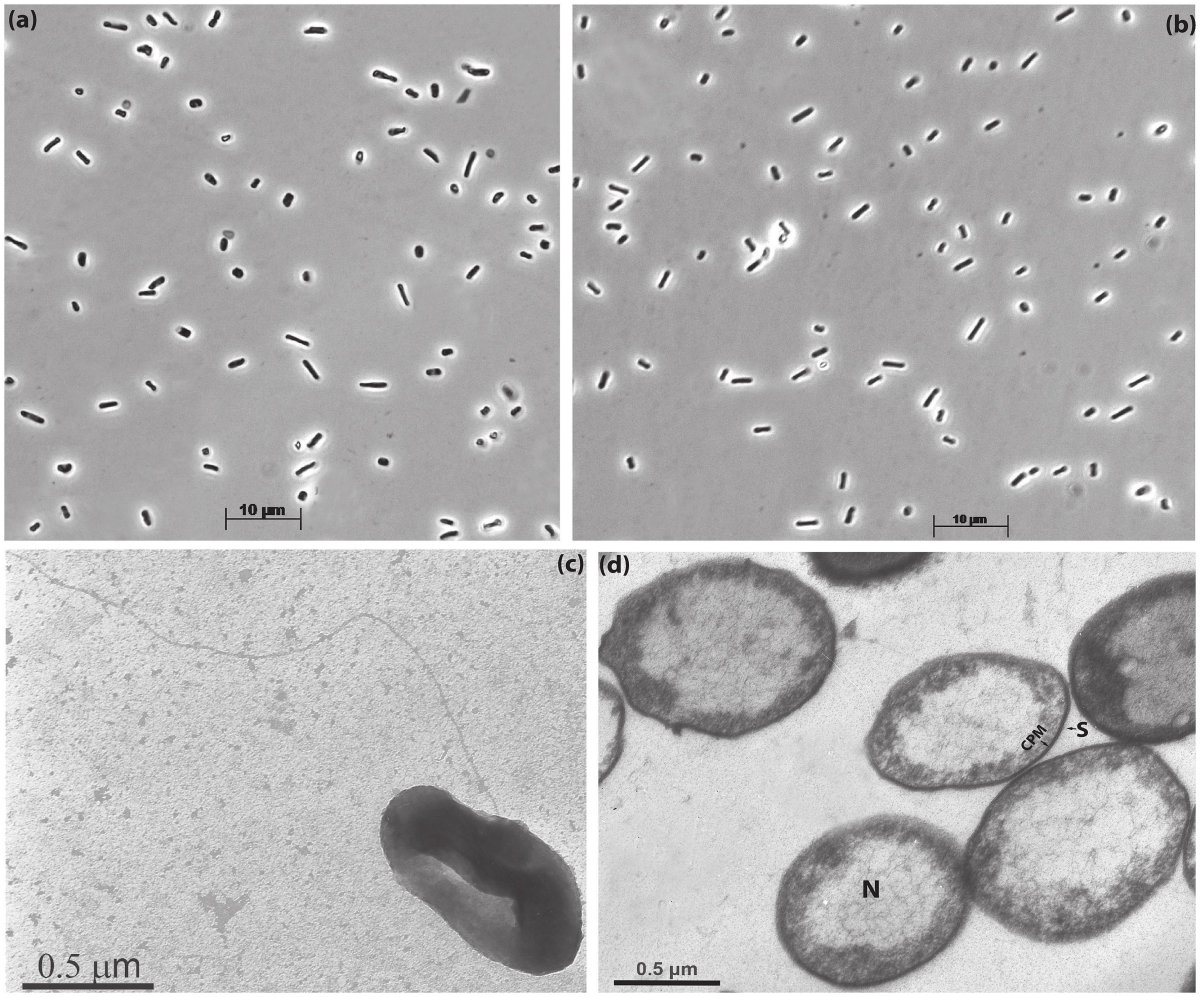
Cell morphology of strains AArcel7^T^ **(a,c,d)** and A-rgal3 **(b)** grown with cellobiose at 4 M total Na^+^ and pH 9.5: **(a,b)** phase contrast microphotographs; **(c)** transmission electron microscopy of the whole cell showing flagellation and **(d)** thin-section electron microscopy showing *S*-layer thin cell wall (S) and extended nucleoid (N). CPM, cytoplasmic membrane.

Despite close relation, the two isolates were clearly different in their macromorphology: AArcel7^T^ formed compact colonies and the cell biomass had an intensive red color, typical for haloarchaea, while A-rgal3 colonies were spreading and the pigmentation was only slightly pink (Supplementary Figure S2). In contrast, the cell micromorphology of these isolates was more similar, with domination of short, flattish motile rods and coccoids (Figures 1a,b). Electron microscopy of AArcel7^T^ cells grown on cellobiose showed the presence of a single thin archaellum and ultrastructural organization typical for many other haloarchaea, with a thin *S*-layer type of the cell wall and enlarged nucleoid (Figures 1c,d). The polar lipid and menaquinone composition of strain AArcel7^T^, determined previously (Bale et al., 2019), was also common for natronoarchaea, with a domination of C20-C20 archaeol and C20-C25 extended archaeol (in equal proportion) as a core and phosphatidylglycerol (PG) and phosphatidylglycerophosphate methyl ether (PGP-Me) as polar heads. The major menaquinone was MK8:8 (84%) and a less abundant MK8:7 (16%).

Growth tests showed that both isolates are obligately aerobic saccharolytics. From the tested polysaccharides, two beta-linked ones supported vigorous growth: insoluble chitin (beta-1,4-linked N-acetylglucosamine residues) and laminarin (beta-1,3/1,6-linked glucose residues). Strain AArcel7^T^ also grew on xylans (birch and beech) and arabinoxylan, while A-rgal3 showed only a weak growth with these substrates. Furthermore, soluble xyloglucan and beta-glucan from barley also weakly stimulated the growth of both isolates in comparison to controls. In addition, both strains grew well with two alpha-1,4-glucans—soluble starch and pullulan. AArcel7^T^ was more versatile in its utilization of sugars, but, in general, both can be considered as polytrophic generalists (Table 1). We also included in substrate profiling the *Natrarchaeobius* strain AArcht7, as a potential new species (according to phylogenomic analysis). Indeed, the results showed that it was different from other AArcht strains and the new isolates AArcel7^T^ and A-rgal3 in its extremely narrow substrate specialization, being able to grow actively only with chitin and its monomer (Table 1).

**Table 1:** Comparative properties of strains AArcel7^T^, A-rgal3, and members of the genus Natrarchaeobius^*^.

Property	AArcel7^T^	A-rgal3	*Natrarchaeobius chitinivorans*		*Natrarchaeobius halalkaliphilus* AArcht-Sl^T^
			AArcht4^T^	AArcht7	
Phenotypic properties					
Cell morphology	Polymorphic rods and coccoids, motile		Dimorphic: flat motile rods on sugars; nonmotile cocci on chitin		
Colonies on amorphous chitin agar	Compact with large chitin clearance, pale	Spreading with small chitin clearance, pale	Compact, with large chitin clearance, pink		Compact, with large chitin clearance, pale
Colonies on amorphous cellulose agar	Compact with small clearance, pink	No growth	No growth		No growth
Utilized polysaccharides (apart from chitin)	Laminarin, xyloglucan (w), xylan, arabinoxylan, barley beta-glucan (w), galactan (w), starch, pullulan	Laminarin, xylan (w), barley beta-glucan (w), starch, pullulan	None		
Utilized sugars	glucose, fructose, sucrose, mannose, raffinose, Naga^**^, glucosamine, lactose, fucose, cellobiose, maltose, trehalose, melibiose, melezitose, xylose (w), galactose (w), rhamnose (w)	mannose, raffinose, cellobiose, maltose, trehalose, melibiose, melezitose, xylose (w), galactose, glucosamine, Naga	sucrose, maltose, trehalose, melezitose, cellobiose, Naga, glucosamine	Naga	Sucrose, maltose, fructose, trehalose, melezitose, Naga, glucosamine
Other carbon sources	Glycerol, pyruvate	Glycerol, pyruvate	Glycerol	Glycerol (w)	Glycerol
Urea as the N-source^***^	+	+	−	+	+
Indol from tryptophane (genomic)^****^	−	−	+	+	−
Salinity range (M total Na^+^)	2.5–4.5 (3.5)	2.5–4.5 (3.5)	3.0–5.0 (4.0)	2.5–4.75 (3.5)	3.0–5.0 (3.5)
Minimal Cl^−^ requirement (M)	2.0	1.5	1.0	1.0	1.5
pH range (optimum)^*****^	7.1–9.8 (8.5–9.0)	7.8–9.9 (9.2–9.5)	7.0–10.0 (9.1–9.3)	7.2–10.1 (9.5)	6.5–9.5 (8.0–8.5)
Maximum growth temperature (°C)	50	47	50	45	55
General genomic properties					
Genome size (Mbp)	4.8	5.3	4.6	4.6	3.5
G + C content (%, genomic)	62.8	63.0	61.9	64.0	61.1
GH18 endo-chitinase genes	3	3	7	11	6
GH20 β-N-acetylhexosaminidase genes	0	0	1	1	0
GH3 β-N-acetylhexosaminidase genes	1	1	1	1	1
Isolation source	Hypersaline alkaline lakes, Wadi an Natrun (Egypt)	Hypersaline soda lakes, southwestern Siberia	Soda crystallizer, southwestern Siberia		Hypersaline alkaline Searles Lake, California

^*^The core membrane lipids in type strains are C20-C20 and C20-C25 archaeols with the PG and PGP-Me head groups; ^**^N-acetyl-glucosamine; ^***^AArcel7 and A-rgal3 genomes encode both urease and urea carboxylase (amydolyase) modules; AArcht7 and AArcht-Sl have only the urea carboxylase operon; ^****^Presence of tryptophanase TpnA gene; ^*****^actual pH.

The pH profiling results indicated that AArcel7^T^ and AArcht7 can be considered as facultatively alkaliphilic, starting to grow actively at pH slightly above 7.0 and up to 10, with an optimum between 8.5 and 9.5. In contrast, A-rgal3 is an obligate alkaliphile with a pH range from 8 to 10 and an optimum at 9.2–9.5. These corresponded well to the chemical composition of the lake brines of the origin: the Wadi an Natrun lakes contained more chloride than carbonates (maximum carbonate alkalinity around 1 M), while the Kulunda Steppe lakes are dominated by sodium carbonates (up to 5 M alkalinity). In their salt profiles, the strains were similar, representing typical extreme halophiles with the range of total Na^+^ from 2.25–2.5 to 4.75 M and an optimum at 3.5–4 M.

### General genome characteristics

Genome of the strain A-rgal3 was assembled into 27 contigs with a total size of 5.16 Mbp and G + C content of 62.9%. Completeness and contamination of the assembly were 99.07 and 0.93%, respectively. According to PGAP annotation, the genome contained 4,721 protein-coding genes, 52 tRNA genes, an operon of rRNA genes (5S, 16S, and 23S), 2 ncRNA genes, and 65 pseudogenes. The genome of strain AArcel7^T^ had similar characteristics (Supplementary Table S1). Identities between 16S rRNA gene sequences from Sanger sequencing and from genome assembly were 99.9% for both strains. Genome statistics for strain AArcht7 were reported previously (Sorokin et al., 2019).

### Phylogenetic analysis and genome-based comparisons

**Figure 2:**
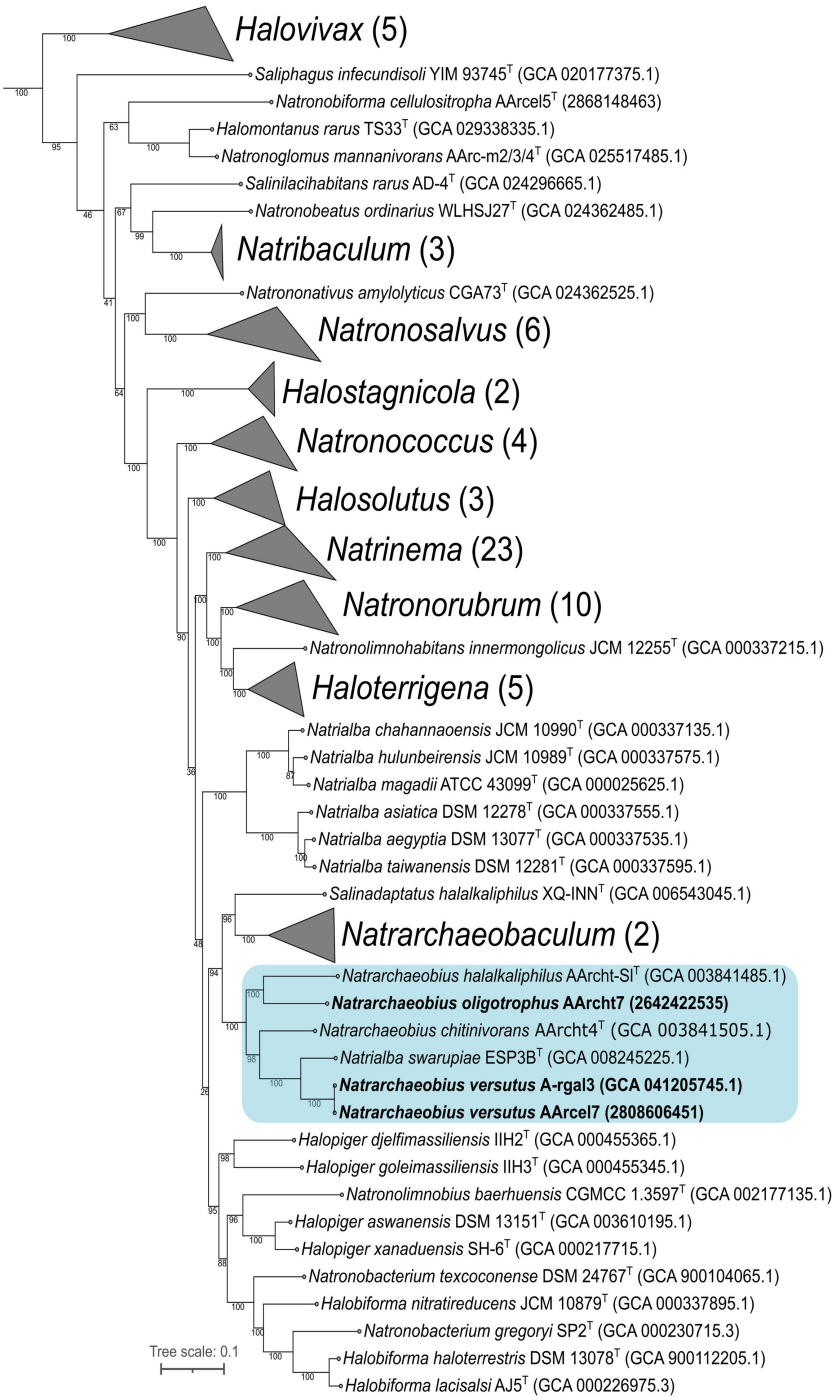
Maximum-likelihood phylogenetic tree based on the “ar122” set of archaeal proteins revealed the position of strains AArcel7 and A-rgal3, and AArcht7 (in bold font) within *Natrialbaceae* family. The numbers at nodes indicate the percentage values of rapid bootstrap (from 1,000 replicates). The blue box indicates the *Natrarchaeobius* genus. *Methanothermobacter thermautotrophicus* DeltaH (GCA 000008645.1), *Archaeoglobus fulgidus* DSM 4304 (GCA 000008665.1), and *Methanocella paludicola* SANAE (GCA 000011005.1) were used as the outgroup (not shown).

According to the results of 16S rRNA gene BLAST search against the core_nt database, closest relatives for both novel isolates were *Natrialba swarupiae* ESP3B_9^T^ (98.85% sequence identity) and different strains of *Natrarchaeobius chitinivorans* (sequence identities of 95.79–96.39%). Maximum likelihood (ML) tree based on comparison of 16S rRNA gene sequences of all type species of *Natrialbaceae* family revealed that strains AArcel7^T^ and A-rgal3 formed a separate cluster between *Natrialba* and *Natrarchaeobius* genera, although support values at key nodes were low (Supplementary Figure S1). At the same time, ML tree based on the “ar122” set of conserved archaeal proteins showed that new isolates clustered together with species of the *Natrarchaeobius* genus, while the remaining species of *Natrialba* formed a separate clade (Figure 2). Therefore, AArcel7^T^ and A-rgal3 should be classified as a novel species within the genus *Natrarchaeobius*. In addition, our results demonstrated that *Natrarchaeobius* strain AArcht7, previously described within the type species *Nar. chitinivorans* likely belong to a different species.

Calculated ANI values between AArcel7^T^/A-rgal3 and other *Natrarchaeobius* species were 78.7–81.3%/78.6–81.2% (Supplementary Figure S3), while AAI values were 74.6–78.0%/74.7–78.1% (Supplementary Figure S4), which are much lower than proposed thresholds of 95% for species delineation in both metrics (Kim et al., 2014; Konstantinidis and Tiedje, 2005). Our results also revealed that AArcel7^T^ and A-rgal3 are strains of the same species (99.4% ANI, 99.3% AAI, 94.5% dDDH on the second formula). Additionally, ANI, AAI, and dDDH values between *Nar. chitinivorans* AArcht4^T^ and AArcht7 were 80.9, 77.2 and 26.1%, which are lower than the proposed border for species differentiation.

### Functional genome analysis

**Figure 3:**
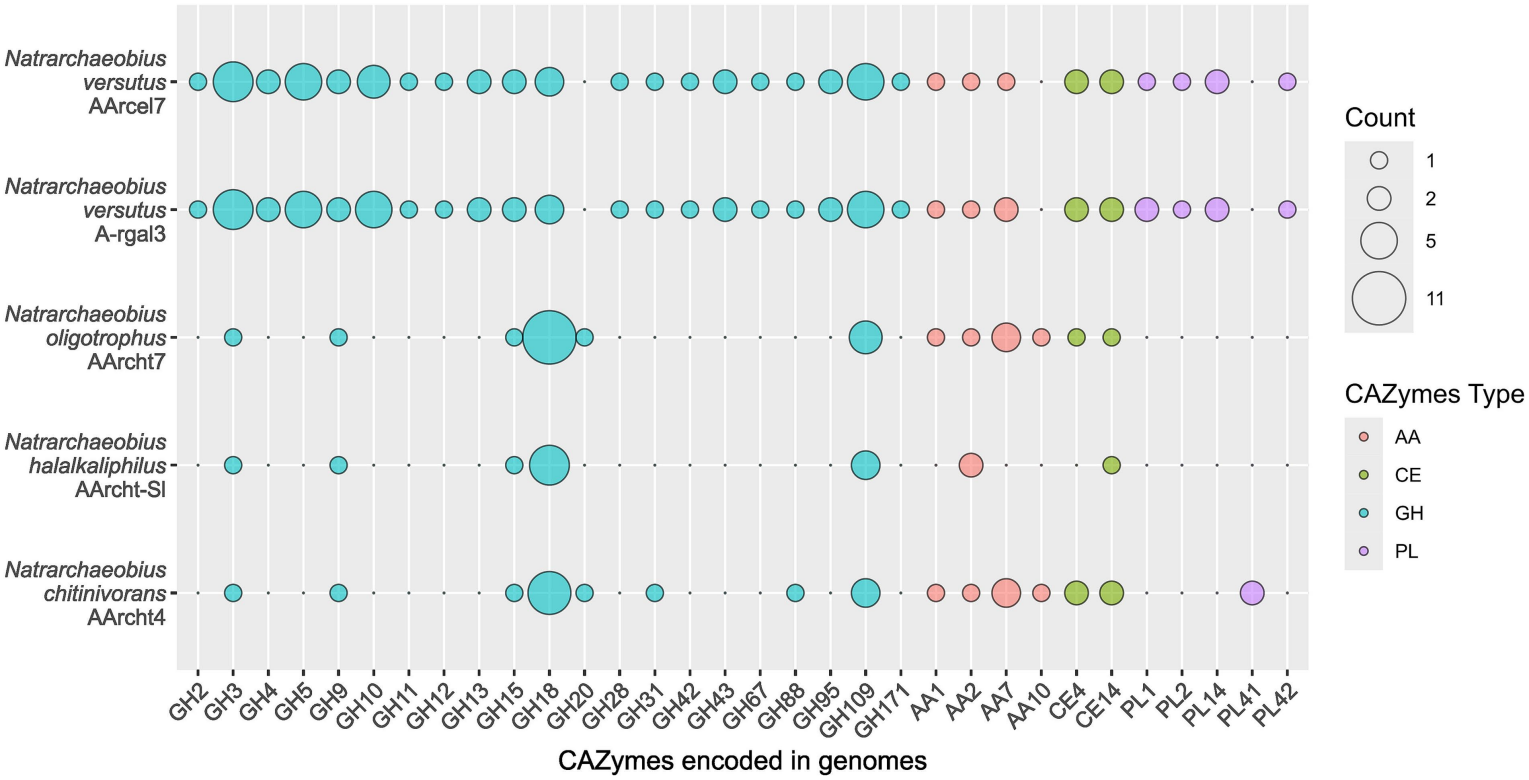
Genes of carbohydrate-active enzyme families found in the genomes of *Natrarchaeobius* species.

Previously, genomes of described species of the *Natrarcheobius* were only briefly analyzed (Sorokin et al., 2019), while the genome of strain AArcel7^T^ was analyzed with a focus on cellulose metabolism only (Elcheninov et al., 2023). Here we analyzed all available genomes of *Natrarcheobius,* including novel isolates AArcel7^T^ and A-rgal3 (five in total). Genomes of the previously described strains of *Natrarchaeobius* (AArcht-Sl, AArcht4, and AArcht7) encoded a relatively small set of glycoside hydrolases (GHs), mostly consisting of endo-chitinases from the GH18 (6–11 genes), β-N-acetylhexosaminidases from the GH20 and the GH3 families (Figure 3). Additionally, endo-β-1,4-glucanase from the GH9 family and hydrolases from the GH109 family with various potential activities were encoded in these genomes. AArcel7^T^ and A-rgal3 also have genes encoding enzymes from GH3, GH18, GH9, and GH109 families. On the other hand, their CAZymes sets are broader, including endoglucanase (GH12), alpha-amylases (GH13, GH15), endo-beta-xylanases (GH10, GH11), endo-β-1,4-mannosidases (GH5 subfamilies 7 and 41), polygalacturonidases (GH28), exo-beta-1,4-N-acetylmuramidase (GH171), and some other polysaccharide hydrolases (Figure 3). In addition to glycoside hydrolases, genomes of *Natrarchaeobius* strains encode a narrow set of carbohydrate esterases (CEs) from CE4 and CE14 families. Most of them presumably have deacetylase activity against N-acetylglucosamine-containing compounds. Enzymes with Auxiliary Activities (AAs) were encoded in the genomes as well. The studied archaeal species have enzymes from AA2 (various peroxidase families). In addition, genomes of AArcel7^T^, A-rgal3, AArcht4^T^, and AArcht7 contained genes of AA1 (multicopper oxidases), AA7 (putative polysaccharide oxidases, so far only known in fungi, according to CAZy). Furthermore, the genomes of strains AArcht4^T^ and AArcht7 encode a homolog of the AA10 family lytic polysaccharide monooxygenases, which can depolymerize crystalline chitin and cellulose chains, producing oxidized products that require a separate pathway to be metabolized (Jiang et al., 2022). This corroborates the observed ability of these archaea to grow on crystalline chitin, albeit much slower than with the amorphous form (Sorokin et al., 2015, 2019). It was also found that strains AArcel7^T^ and A-rgal3 possess genes encoding various polysaccharide lyases (Figure 3): putative pectate lyases from PL1 and PL2 families, putative alginate lyase (PL14), and a L-rhamnose-alpha-1,4-D-glucuronate lyase (PL42). Notably, the gene of alginate lyase from the PL41 family was exclusively identified in the genome of strain AArcht4^T^, and seems to be absent in the genomes of strains A-rgal3 and AArcel7. Moreover, it was demonstrated that strain A-rgal3 possesses the capacity to grow on rhamnogalacturonan, or at least to be able to hydrolyze certain polysaccharides containing uronic acid residues.

**Figure 4:**
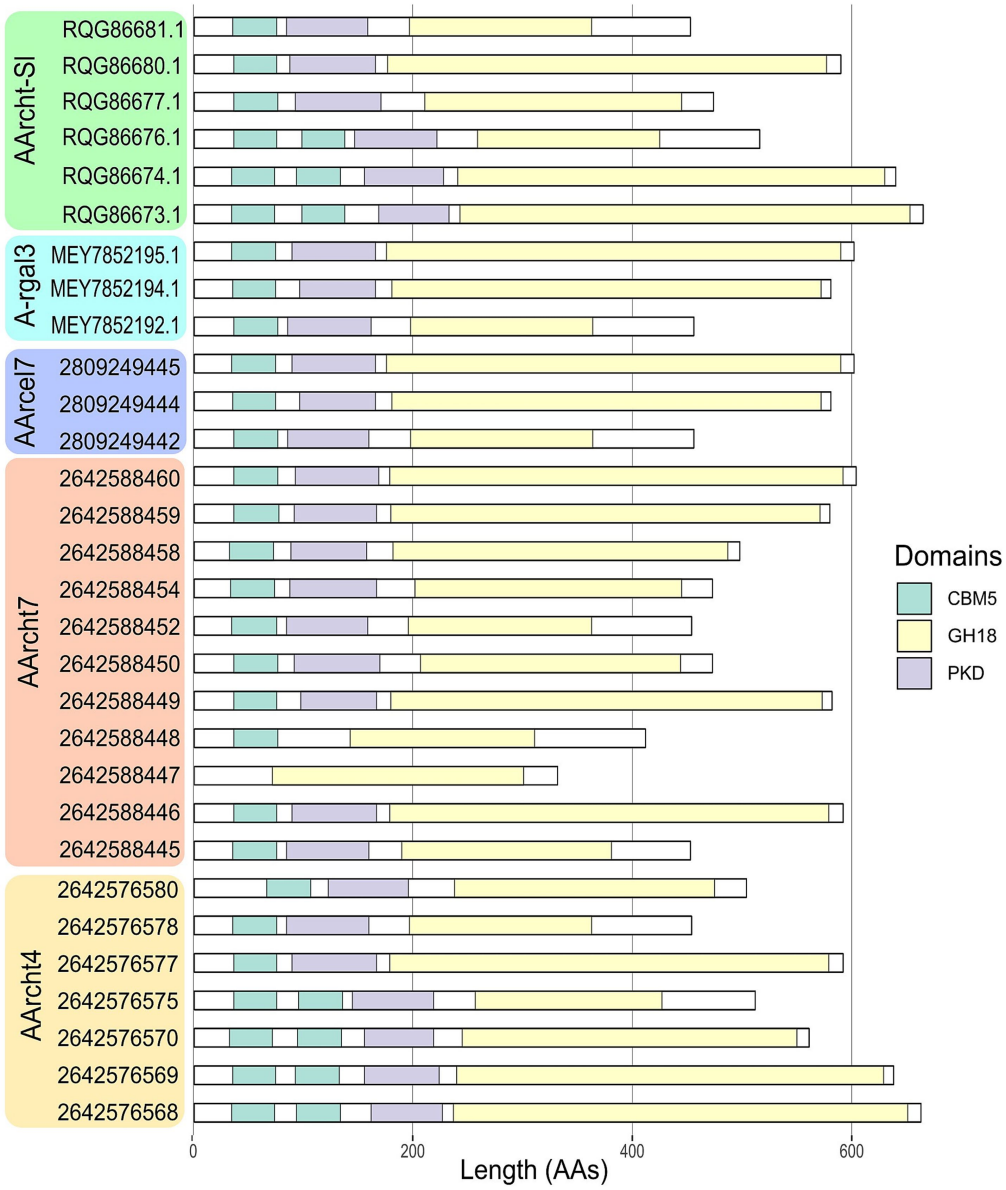
Domain organization of chitinases, found in the genus *Natrarchaeobius*. CBM5 (Carbohydrate-Binding Module family 5), GH18 (catalytic domain of chitinase from Glycoside Hydrolase family 18), PKD (Polycystic Kidney Disease-like domain).

A comprehensive study of all the chitinases encoded in the *Natrarchaeobius* genomes revealed variations in their domain architecture. For instance, the number of substrate-binding domains (CBM5 and PKD) varied among the studied proteins. Typically, this variation leads to the presence of multiple domains, except that the GH18 from AArcht7 (2642588447) lacked any substrate-binding domains. It is noteworthy that variation in the size of the catalytic domains has been observed in certain chitinase sequences as well. According to HMMER results, some GH18 sequences possess domains of a substantially reduced length (approximately twofold shorter). This variation has been observed not only between chitinases from different *Natrarchaeobius* species, but also between chitinases encoded in the same genome (Figure 4). Since chitin, as well as cellulose, exists in several allomorphic forms (e.g., *α*-, *β*-, *γ*-chitin) with distinct degree of crystallization (Salmon and Hudson, 1997), it is conceivable that the observed multiple isoforms of chitinases, which exhibit variation in the number of chitin-binding modules, are necessary for the hydrolysis of all these allomorphs. This observation is consistent with the findings that these binding domains are predominantly active against highly crystalline regions of the chitin polymer (Hashimoto et al., 2000).

**Figure 5:**
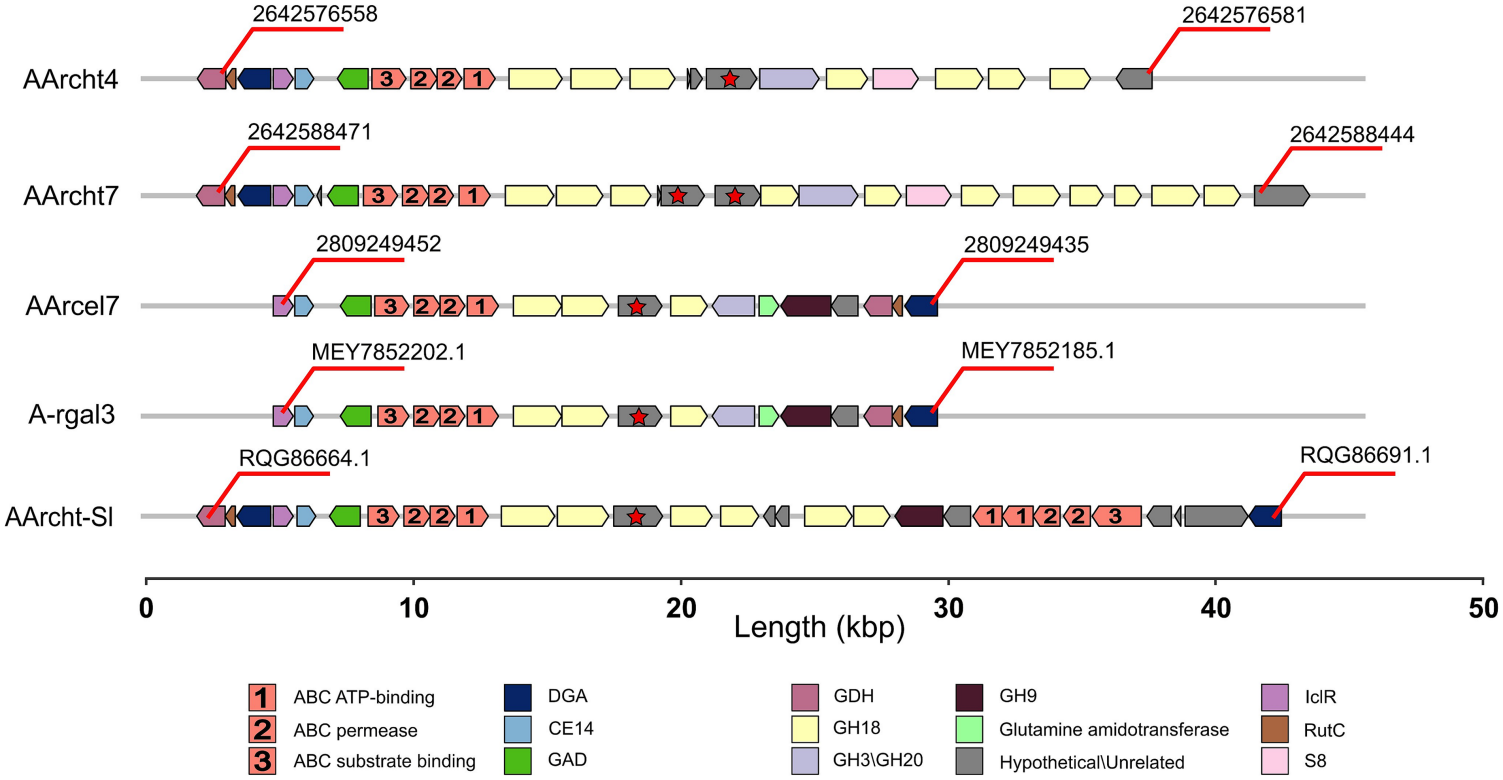
Gene clusters encoding chitinases in the genomes of *Natrarchaeobius* species: DGA (putative D-glucosaminate-6-phosphate ammonia lyase), GAD (galactonate dehydratase), GDH (glucose dehydrogenase), IclR (HTH-type transcriptional regulator from the IclR family), RutC (transcriptional repressor from the RutC family), S8 (serine peptidase from the S8 family). Hypothetical genes marked with red stars indicate the presence of the ambiguous chitinase.

**Figure 6:**
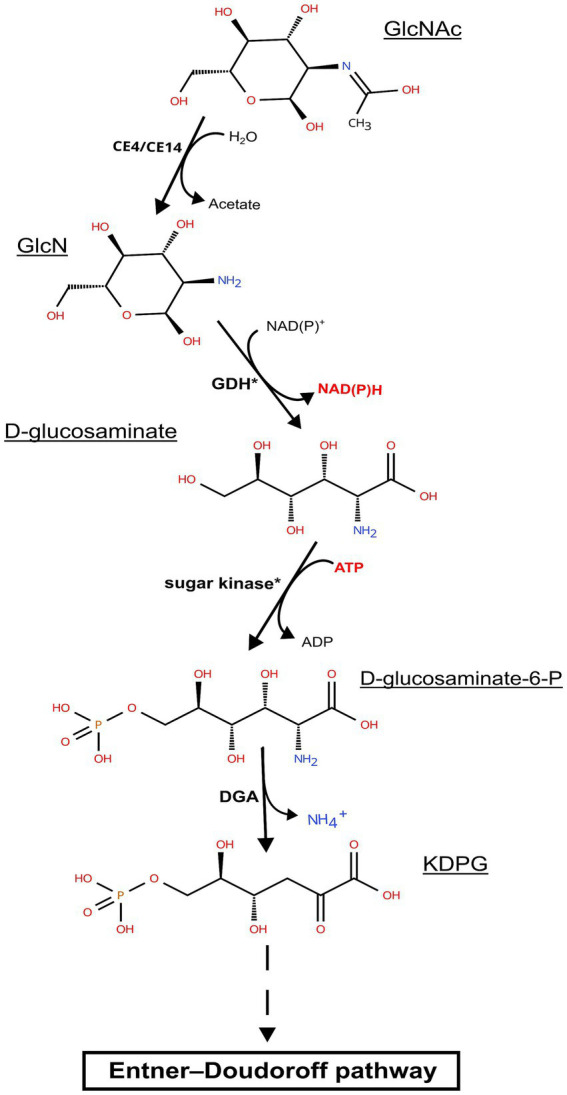
Predicted pathway of N-acetylglucosamine metabolism in representatives of the genus *Natrarchaeobius*: CE4/CE14, carbohydrate esterase from CE4/CE14 family; GDH, glucose dehydrogenase; DGA, putative D-glucosaminate-6-phosphate ammonia lyase; GclNAc, N-acetylglucosamine; GlcN, D-glucosamine; KDPG, 2-keto-3-deoxy-6-P-gluconate. The asterisks indicate that target enzymes are not characterized; however, there may be homologs having broad substrate specificity that perform these reactions.

The chitinase genes are located in large clusters in *Natrarchaeobius* species (Figure 5). In addition to chitinases, these clusters encode ABC transporters, substrate-binding subunits of which were homologous to characterized diacetylchitobiose-binding proteins in the ABC transporter of *Streptomyces coelicolor* (Saito et al., 2007) capable of growing on chitin. This allows us to assume that these transporters could participate in the import of chitin oligo- and monomers into the cells. Furthermore, all clusters encode a single copy of the enzyme belonging to the CE14 family with a known function as of N-acetylglucosamine (GlcNAc) deacetylase. Enzymes from the GH3 and/or GH20 families were also encoded in these gene clusters (except for the genome of AArcht-Sl). In the genome assemblies of AArcel7, A-rgal3, and AArcht-Sl, there was an occurrence of a GH9 gene. These clusters in each genome also contain one or two genes encoding proteins with chitin-binding domains but lacking the catalytic domain of the GH18 chitinase family. It is noteworthy that genes encoding several other enzymes were located near chitinase genes, which might play a role in the further GlcNAc metabolism, not yet recognized in chitin-utilizing haloarchaea. Only for the nanohaloarchaeon “*Ca.* Nanohalobium constans” associated with the chitinotrophic haloarchaeon *Halomicrobium* sp., the pathway of GlcNAc catabolism was predicted (La cono et al., 2020). In the *Natrarchaeobius* species, we can predict at least a partial pathway (Figure 6). In the first step, diacetylchitobiose or GlcNAc is deacetylated by CE14 carbohydrate esterase, forming D-glucosamine (GclN). Next, broad-specificity glucose 1-dehydrogenase can oxidize GclN to D-glucosaminate (Bonete et al., 1996). The latter is probably phosphorylated by an unknown carbohydrate kinase to D-glucosaminate-6-phosphate, which is converted to 2-dehydro-3-deoxy-6-phosphogluconate (KDPG) by an enzyme homologous to D-glucosaminate-6-phosphate ammonia lyase (Phillips et al., 2021). Finally, KDPG is metabolized in the Entner-Doudoroff pathway. Gene expression in these clusters is regulated by an HTH-type transcriptional regulator from the IclR family, responsible for activation of sugar catabolizing genes in other haloarchaea (Johnsen et al., 2015), and a transcription repressor from the RutC family (Figure 5). However, to confirm this hypothetical pathway, additional biochemical and omics-based studies are required.

Genomic analysis has also revealed several noteworthy differences between the AArcht7 and AArcht4^T^ strains. For instance, we have identified genes encoding homologs of all the key enzymes from the pentose bisphosphate pathway, which links metabolism of nucleosides with the central metabolism, within the AArcht7 genome assembly. Conversely, the genome assembly of the AArcht4^T^ encoded enzymes involved in the non-carboxylating pentose bisphosphate pathway (with the exception of glycolaldehyde reductase), a feature more commonly associated with the haloarchaea (Sato et al., 2022).

It is well known that chitin, despite its abundance, does not seem to accumulate in natural environments with neutral pH (Gooday, 1990). In soda lakes, massive populations of brine shrimp from the genus *Artemia* and from “soda flies” *Ephydridae* contribute to high chitin production (Dana et al., 1990; Mengistou, 2016). This polymer is efficiently degraded by natronophilic prokaryotes in sediment (Sorokin et al., 2012). Given these observations, the primary role of chitin decomposition in soda lakes is likely carried out by natronophilic species from the *Archaea* and *Bacteria* domains, which incorporate carbon and nitrogen from the polymer into biogeochemical cycles in these extreme habitats. However, despite the confirmed presence of anaerobic and aerobic natronophilic chitinotrophic species in both domains (Sorokin et al., 2012, 2014, 2015, 2016, 2019), ecological aspects of chitin degradation in soda lakes remain poorly understood and still to be elucidated.

## Conclusion

In this study, we present a detailed characterization of two strains of alkaliphilic, extremely halophilic archaea isolated from hypersaline alkaline lakes in Wadi an Natrun (Egypt) and the Kulunda steppe (Russia). Strains AArcel7^T^ and A-rgal3 are aerobic heterotrophs capable of utilizing mono saccharides and polysaccharides, including chitin. Phylogenetic analyses based on comparison of 16S rRNA gene sequences and conserved protein sequences placed the new isolates into the genus *Natrarchaeobius* as a separate species. In contrast to previously described *Natrarchaeobius* chitin-specialized species, novel strains possessed more versatile metabolism, utilizing a wider carbohydrate spectrum. Moreover, further investigation of the genomes of these archaea has enabled the reconstruction of a putative metabolic pathway for N-acetylglucosamine utilization, which potentially allows members of this genus to thrive on chitin. Additional phenotypic and genomic characterisation and a significant phylogenetic divergence of strain AArcht7, previously classified as a member of the type species of the genus *Natrarchaeobius*, also allowed us to propose reclassifying it as a separate species within this genus.

On the basis of phylogenetic analysis and distinct phenotypic properties, two extremely natronoarchaeal strains AArcel7 and A-rgal3 are proposed to be classified as members of a novel species of the genus *Natrarchaeobius*, *Nar*. *versutus* sp. nov. (type strain AArcel7^T^), while strain AArcht7^T^ is suggested to be reclassified as *Nar. oligotrophus*.

## Description of *Natrarchaebius versutus* sp. nov.

[ver.su’tus] L. masc. adj. *versutus*, versatile

The species description is based on two closely related isolates, strains AArcel7^T^ and A-rgal3. The cells are motile, flattened rods or coccoids, 0.5–0.8 × 1 to 3 μm, with a thin monolayer cell wall. Cell biomass grown with sugars is pink-orange. Colonies of strain AArcel7^T^ on amorphous chitin agar are compact, orange, and form a large clearance zone, while strain A-rgal3 formed pale spreading colonies with a limited chitin clearance. AArcel7^T^ is also forming colonies on amorphous cellulose agar with a limited cellulose clearance, but it cannot utilize cellulose as a growth substrate in liquid culture. The core membrane lipids in AArcel7^T^ are represented by archaeol C_20_-C_20_ and extended archaeol C_20_-C_25_ DGE (in equal proportions) with the polar heads as phosphatidylglycerophosphate methylester (PGP-Me) and phosphatidylglycerol (PG). A glycolipid is also detected, but as a minor component (<1% of the total). Similar to the already described species of *Natrarchaeobius*, both isolates are strictly aerobic saccharolytic heterotrophs, growing best with amorphous chitin as a carbon and energy source. However, both can also utilize a range of other beta-glucans, including laminarin, xylan and barley beta-glucan (weakly); and alpha-glucans including starch and pullulan. The utilized sugars by both strains included mannose, raffinose, trehalose, cellobiose, maltose, melibiose, melezitose, galactose, glucosamine, and N-acetylglucosamine, and xylose (weak). In addition, AArcel7^T^ can also grow with glucose, fructose, sucrose, lactose, and fucose. Proteolytic and lipolytic activities are absent. The nitrogen source is ammonium and urea. High Mg is not required for growth. Indole is not formed from tryptophan. Extremely halophilic, growing optimally at 3.5 M total Na^+^ and alkaliphilic with the pH range for growth between 7 and 9.9 (optimum between 8.5 and 9.5). The maximum growth temperatures (at pH 9) are 47–50°C. The G + C content of the genomic DNA in the type strain is 62.8 mol% (genome). The habitat is hypersaline soda lakes. The type strain AArcel7^T^ (DSM 119677 = UNIQEM U973^T^) was isolated from an oxic sediment-brine mix sample of hypersaline alkaline lakes in Wadi an Natrun (Egypt). Strain A-rgal3 (UQM 41910) was enriched from an oxic sediment-brine mix sample of hypersaline lakes in Kulunda Steppe (Altai region, Russia).

## Description of *Natrarchaeobius oligotrophus* sp. nov.

[o.li.go.tro’phus] Gr. masc. adj. *oligos*, little; Gr. masc./fem. adj. *trophos*, a feeder, rearer, that which nourishes; N.L. masc. adj. *oligotrophus*, utilizer of few substrates, oligotrophic

The species description is based on a single strain, AArcht7 previously classified within the *Natrarchaeobius chitinivorans* species. Colonies on amorphous chitin agar are orange, convex, and smooth, forming a large clearance around (Supplementary Figure S5a). The cells are motile, flattish rods or nonmotile coccoids (on chitin), 0.5–1.0 × 1–3 μm (Supplementary Figure S5b). In contrast to other *Natrarchaeobius* strains, it is extremely narrow and specialized, actively growing only on chitin and N-acetylglucosamine. The inorganic nitrogen source is ammonium and urea (during growth on glycerol). High Mg is not required for growth. Indole formation from tryptophan is positive. Extremely halophilic, growing optimally at 3.5 M total Na^+^ and alkaliphilic with the pH range for growth between 7.2 and 10.1 (optimum at 9.5). The maximum growth temperature (at pH 9.5) is 45°C. The G + C content of the genomic DNA in the type strain is 64.0%. The type strain is AArcht7^T^ (DSM 119936 = UNIQEM U967), is isolated from a trona crystallizer pond in Kulunda Steppe (Altai region, Russia).

## Data Availability

The datasets presented in this study can be found in online repositories. The names of the repository/repositories and accession number(s) can be found in the article/Supplementary material.
