# Supplementary material for: Natrarchaeobiusversutus sp. nov. and Natrarchaeobius oligotrophus sp. nov., chitinotrophic natronoarchaea from hypersaline soda lakes, and functional genome analysis of the Natrarchaeobius species
Source: Front Microbiol. 2025 Jul 30;16:1640521. doi: 10.3389/fmicb.2025.1640521 (PMC12343721; doi:10.3389/fmicb.2025.1640521)
Supplement: Supplementary file 1 [file Data_Sheet_1.pdf]

Supplementary Table S1. Characteristics of genome assemblies of strain AArcel7 and A-rgal3.

Characteristic	AArcel7	A-rgal3
ID	2808606451 (IMG/M)	GCA_041205745.1 (GenBank)
Size, bp	5,121,137	5,163,047
G+C, %	62.8	62.9
Contigs number	23 (22 scaffolds)	27
N50, bp	685,917	446,326
Completeness, %	99.07	99.07
Contamination, %	0.93	0.93
Protein-coding genes	4,769	4,721
rRNA genes	4 (two fragments of 23S rRNA)	3
tRNA genes	44	47
ncRNA genes	3	2
Pseudogenes	na*	65

* – IMG/M does not estimate numbers of pseudogenes

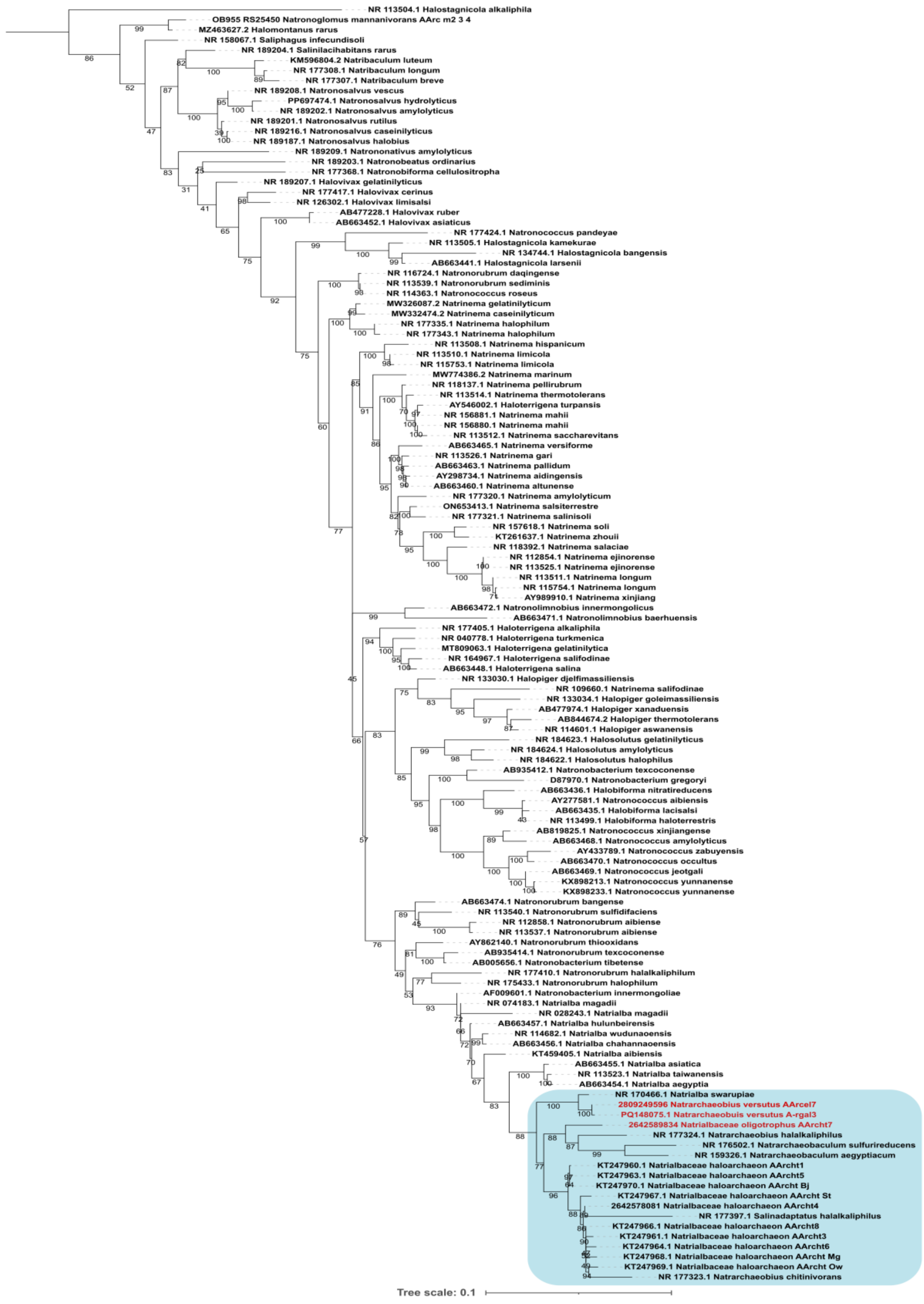

Supplementary Figure S1. Maximum likelihood phylogenetic tree based on comparison of 16S rRNA genes within *Natrialbaceae* family and showing position of strains AArcel7, A-rgal3 and AArcht7 (names marked by red color). Tree construction was performed using IQTree2 v.2.3.5 with TIM+F+R4 model and 1000 UFboot replications. Sequence of *Methanosarcina barkeri* DSM 800 (AB973360) was used as the outgroup (not shown).

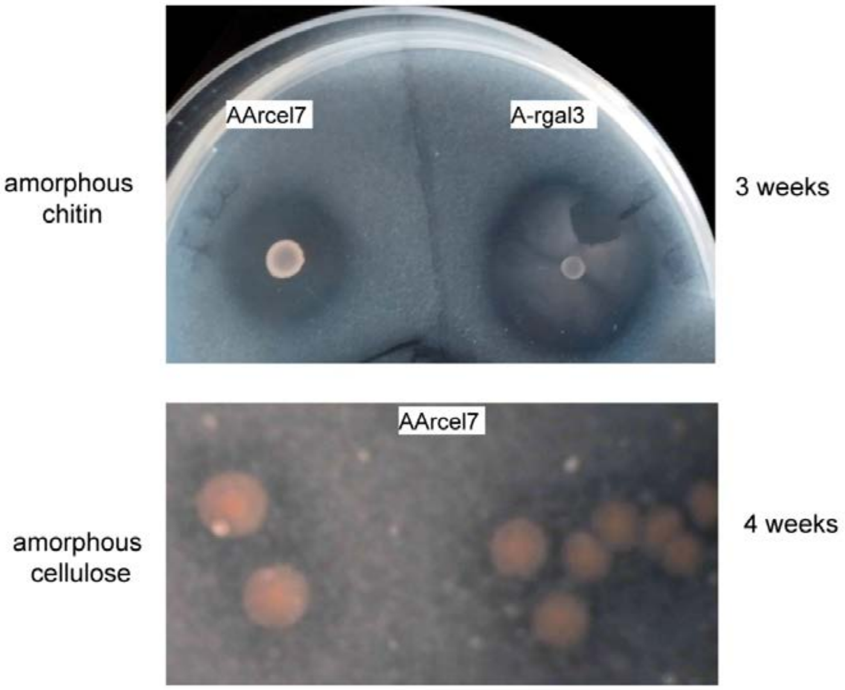

Supplementary Figure S2. Colonies of strains AArcel7 and A-rgal3 on the plate with amorphous chitin (upper half). Colonies of the strains AArcel7 on the plate with amorphous cellulose (lower half).

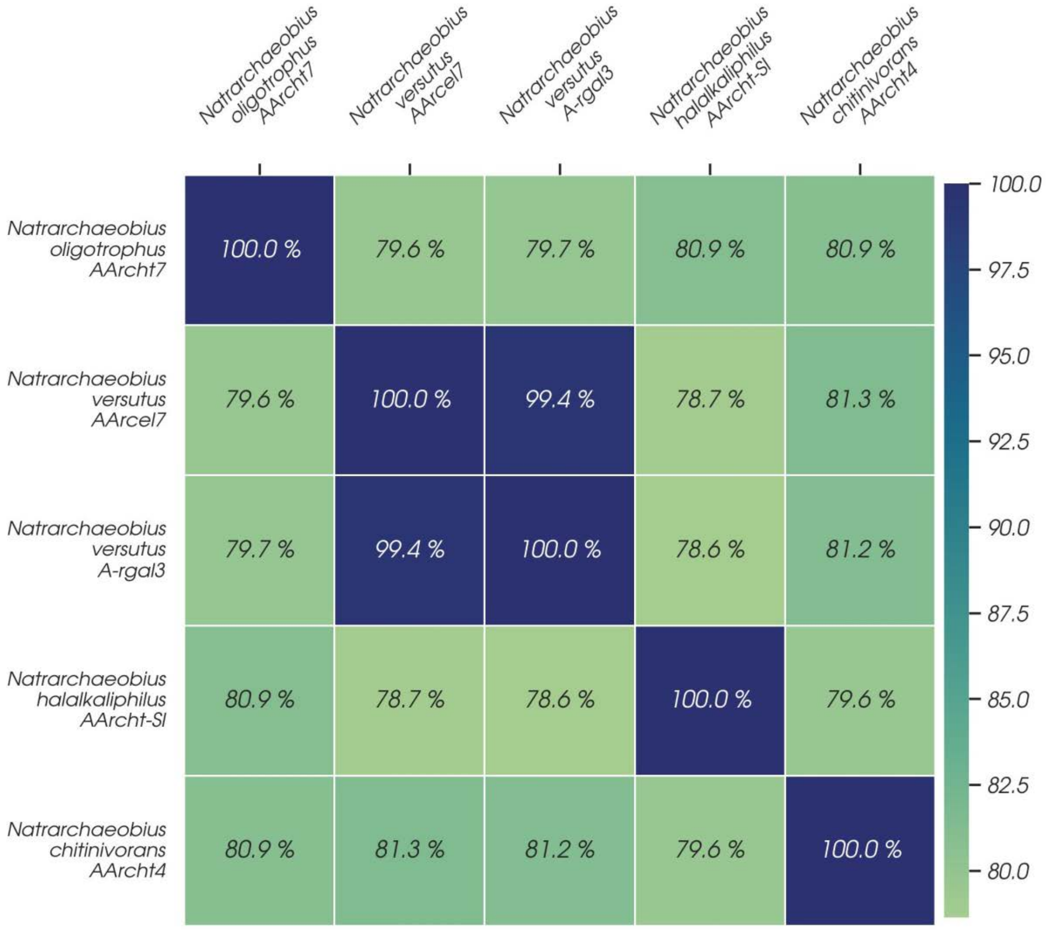

Supplementary Figure S3. Average nucleotide identity values between species of the *Natrarchaeobius* genus.

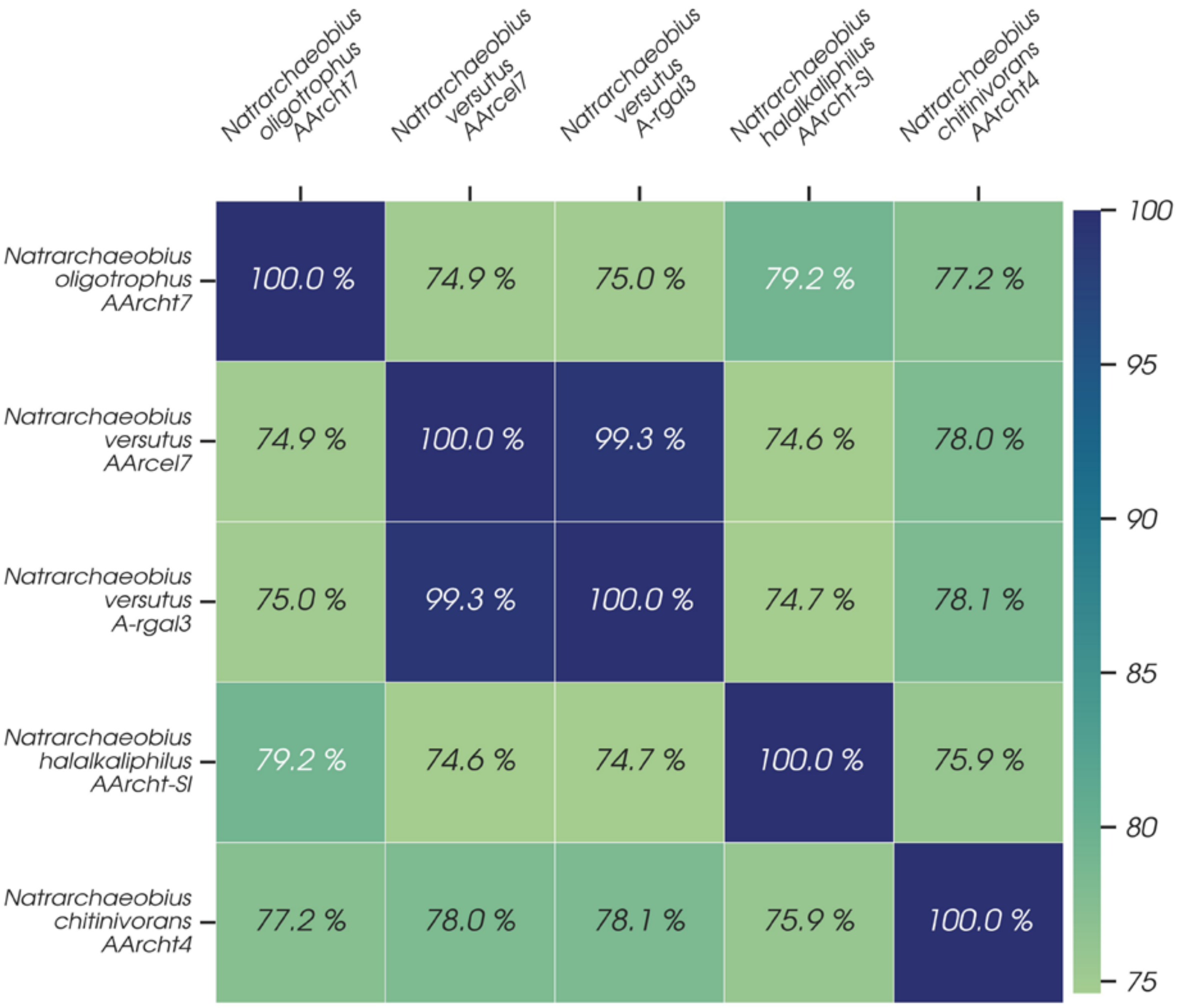

Supplementary Figure S4. Average amino acid identity values between species of the *Natrarchaeobius* genus.

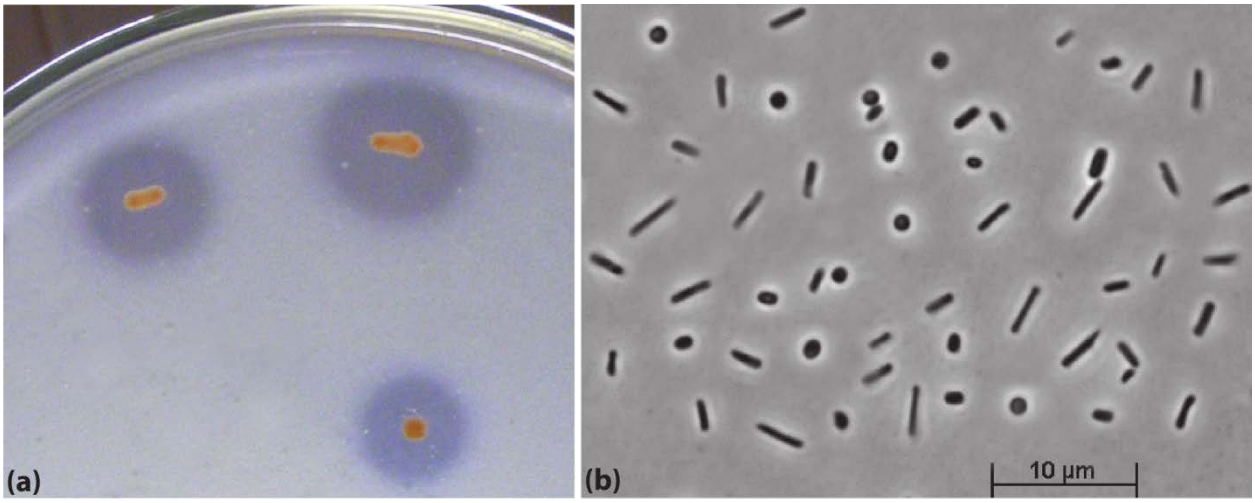

Supplementary Figure S5. a – amorphous chitin-hydrolyzing colonies of strain AArcht7; b – cell morphology of strain AArcht7 grown with amorphous chitin.
